# Association of polyethylene type with revision and dislocation risk following primary total hip arthroplasty: a population-based cohort study from the Danish Hip Arthroplasty Register

**DOI:** 10.2340/17453674.2026.46487

**Published:** 2026-09-07

**Authors:** Marwan CHABAITA, Afrim ILJAZI, Michala Skovlund SØRENSEN, Frank ERIKSSON, Søren OVERGAARD, Michael Mørk PETERSEN

**Affiliations:** 1 Department of Orthopedic Surgery, Copenhagen University Hospital, Rigshospitalet; 2 Department of Clinical Medicine, University of Copenhagen, Copenhagen; 3 Department of Public Health, Section of Biostatistics, Copenhagen University, Copenhagen; 4 Department of Orthopedic Surgery and Traumatology, Copenhagen University Hospital, Bispebjerg-Frederiksberg, Denmark

## Abstract

**Background and purpose:**

Highly cross-linked polyethylene (HXLPE) has been widely adopted to address wear in total hip arthroplasty (THA), yet population-based evidence remains limited. We aimed to evaluate the long-term risk of all-cause revision, dislocation, and cause-specific revision according to polyethylene type in primary THA.

**Methods:**

We conducted a population-based cohort study using data from the Danish Hip Arthroplasty Register and the Danish National Patient Register, including 48,618 patients undergoing primary THA for osteoarthritis from 2000 to 2016. Patients were followed until revision, dislocation, death, year 10, or December 2021. We compared HXLPE with non-HXLPE for patients receiving implants with 28 mm, 32 mm, and 36 mm femoral heads. We estimated standardized risk differences using targeted maximum likelihood estimation comparing polyethylene type stratified by femoral head size and standardized to age, sex, fixation, and Charlson Comorbidity Index.

**Results:**

Standardized risk differences showed favorable results for HXLPE regarding all-cause revision (–3.1%, 95% confidence interval [CI] –4.5 to –1.6), dislocation (–1.9%, CI –3.1 to –0.6), revision due to dislocation (–1.3%, CI –1.9 to –0.7), and revision due to infection (–0.9%, CI –1.3 to –0.4) for 36 mm heads, and for dislocation (–1.4%, CI –2.6 to –0.2) and revision due to aseptic loosening (–0.8%, CI –1.1 to –0.5) for 32 mm heads. There was no difference between HXLPE and non-HXPLE for any outcomes for the 28 mm group.

**Conclusion:**

Among 36 mm implants, HXLPE was associated with lower risk of dislocation, all-cause revision, and revision due to infection and dislocation. Among 32 mm implants, HXLPE was associated with lower risk of dislocation and aseptic loosening revision. These findings may suggest potential advantages of HXLPE, though caution is warranted given the observational design.

Total hip arthroplasty (THA) is a well-established treatment for hip osteoarthritis, but the long-term durability of implants has been limited by material wear and associated complications. Conventional polyethylene (non-HXLPE) has shown considerable wear over time, leading to particle-induced periprosthetic osteolysis and aseptic loosening, which are amongst the most common causes for revision surgery [1-6].

Highly cross-linked polyethylene (HXLPE) has demonstrated a marked reduction in both linear and volumetric wear [7-9]. In theory, this should reduce the incidence of osteolysis and aseptic loosening, thereby improving long-term implant survivorship and reducing the rates of revision.

Despite widespread adoption, the clinical efficacy of HXLPE has largely been assumed based on laboratory data and small cohort studies. Few population-based evaluations have assessed whether HXLPE meaningfully impacts long-term outcomes, more specifically regarding revision and dislocation risk [10,11].

The aim of our study was to compare the risk of all-cause revision and dislocation between HXLPE and non-HXLPE implants following primary THA, stratified by femoral head size. Furthermore, the risk of cause-specific revision for aseptic loosening, infection, and dislocation between HXLPE and non-HXLPE implants was examined, with a minimum follow-up period of 5 years, and a maximum follow-up period of 10 years.

## Methods

### Study design and data sources

This population-based cohort study was based on prospectively collected data from the Danish Hip Arthroplasty Register (DHR) and the Danish National Patient Register (DNPR). The DHR is a nationwide registry that records all THAs, including both primary and revision procedures, performed in public and private hospitals in Denmark, with a registration completeness of approximately 98% for primary THA and 95% for revision procedures [3]. The DNPR is an administrative database containing information on all hospital contacts, including discharge diagnoses. Linkage between the DHR and the DNPR was achieved using Denmark’s unique 10-digit personal identification number, assigned to all residents [12]. The study was conducted in accordance with the Reporting of Studies Conducted Using Observational Routinely Collected Health Data (RECORD) statement [13].

### Study population

The study cohort included all primary THAs performed in Denmark for primary osteoarthritis between January 1, 2000, and December 31, 2016, thereby guaranteeing at least 5 years of potential follow-up (the dataset included patients operated on until December 31, 2021). Eligible patients underwent THA with any polyethylene liner combined with a femoral head of either metal or ceramic in sizes of 28, 32, or 36 mm, and implanted using the posterior approach. Exclusion criteria comprised metal-on-metal implants, dual-mobility implants, constrained liners, and cases with unknown laterality. Patients younger than 40 years were also excluded because primary osteoarthritis is uncommon in this age group, as were individuals who emigrated before their primary surgery. Emigrated patients were excluded due to incomplete registry follow-up, as complications in this group would be managed and recorded outside the Danish healthcare system. For patients who received bilateral THA, only the first operated-on hip was considered. Follow-up continued from the date of the primary THA until death, implant removal, 10 years’ follow-up, or December 31, 2021, whichever occurred first.

### Outcomes

The primary objective was to estimate the 10-year cumulative incidence of all-cause revision in patients with an HXLPE-liner compared with non-HXLPE.

The secondary objectives were to estimate the 10-year cumulative incidence of cause-specific revision for aseptic loosening, infection, and dislocation in patients with an HXLPE compared with non-HXLPE liner, and the 10-year cumulative incidence of dislocation in patients with an HXLPE liner compared with regular PE.

Dislocations were identified in the DNPR using a validated algorithm with a sensitivity of 91% and a positive predictive value of 93% [14] (see Appendix 1). Revision for any cause was identified in the DNPR and defined as the presence of a NOMESCO procedure code indicating removal of a bone-anchored component corresponding to the primary THA. Cause-specific revision was identified in the DHR when a revision was recorded after the primary THA, based on the documented indication for that revision.

### Statistics

All results were presented separately for patients receiving THAs with 28 mm, 32 mm, and 36 mm femoral heads. Continuous variables were presented as means with standard deviations (SD), while categorical variables were presented as number and percentages. Differences between groups in baseline characteristics were tested with ANOVA for continuous variables and χ^2^ test for categorical variables. All analyses were done in R (R Foundation for Statistical Computing, Vienna, Austria).

We present both unstandardized 10-year treatment-specific risks and treatment-specific risks and risk differences standardized to the population distribution of age, sex, fixation, and Charlson Comorbidity Index (CCI). The standardized quantities account for confounding by imbalances in prognostic pre-treatment patient characteristics between the treatment groups. If there is no further unmeasured confounding, the standardized treatment-specific risk estimates the proportion of the population who would experience the event had everyone been treated with the specific polyethylene type and the standardized risk difference estimates the average treatment effect (ATE), the difference in population-level risk had everyone been given non-HXLPE compared with had everyone been given HXLPE.

In both the unstandardized and standardized analyses, death was a competing risk for all-cause revision; death and revision due to other causes were competing risks for cause-specific revision due to aseptic loosening, infection or dislocation; and death and revision were competing risks for dislocation. Potential-follow up was calculated using reverse Kaplan–Meier.

Unadjusted cumulative incidences were calculated with the Aalen–Johansen estimator. Estimation of the standardized risks was performed using targeted maximum likelihood estimation (TMLE) for continuous-time competing risks data as described by Rytgaard et al. [15,16]. TMLE provides consistent estimates under weak assumptions, is doubly robust to model misspecification, and integrates machine learning to flexibly adjust for covariates. We used the concrete package for the TMLE analysis [17]. Treatment was PE type, and the baseline covariates were age, sex, fixation, and CCI. For the treatment mechanism, we employed the Super Learner package with random forests (ranger) and generalized linear models. For the cause-specific and censoring hazards, we specified a set of candidate Cox proportional hazards models (see Appendix 2) and cross-validation was used to select the optimal learners from this library. Standard errors were obtained from the efficient influence curve, and 2-sided 95% confidence intervals (CI) were reported. Standardized cumulative incidences and ATE are reported as estimate followed by the 95% CI in parentheses. We considered non-overlapping confidence intervals for risk differences as statistically significant.

### Ethics, data sharing plan, funding, use of AI, and disclosures

This study was a subsidiary analysis of a larger investigation examining dislocations and multiple revisions following THA. The parent study was approved by the Data Protection Agency of the Capital Region of Denmark (P-2022-717). In Denmark, approval from institutional review boards or regional ethics committees is not required for register-based studies. All data processing was conducted in accordance with the General Data Protection Regulation.

During the preparation of this work the authors used ChatGPT (GPT-5.2, OpenAI) to edit spelling and grammar, as well as to enhance the readability and language of this manuscript. After using this tool, the authors reviewed and edited the content as needed and take full responsibility for the content of the publication.

MC and AI received funding from Rigshospitalet’s Forskningspulje (The Rigshospitalet Research Fund) for this study. Complete disclosure of interest forms according to ICMJE are available on the article page, doi: 10.2340/17453674.2026.46487

## Results

### Baseline characteristics

**Figure 1:**
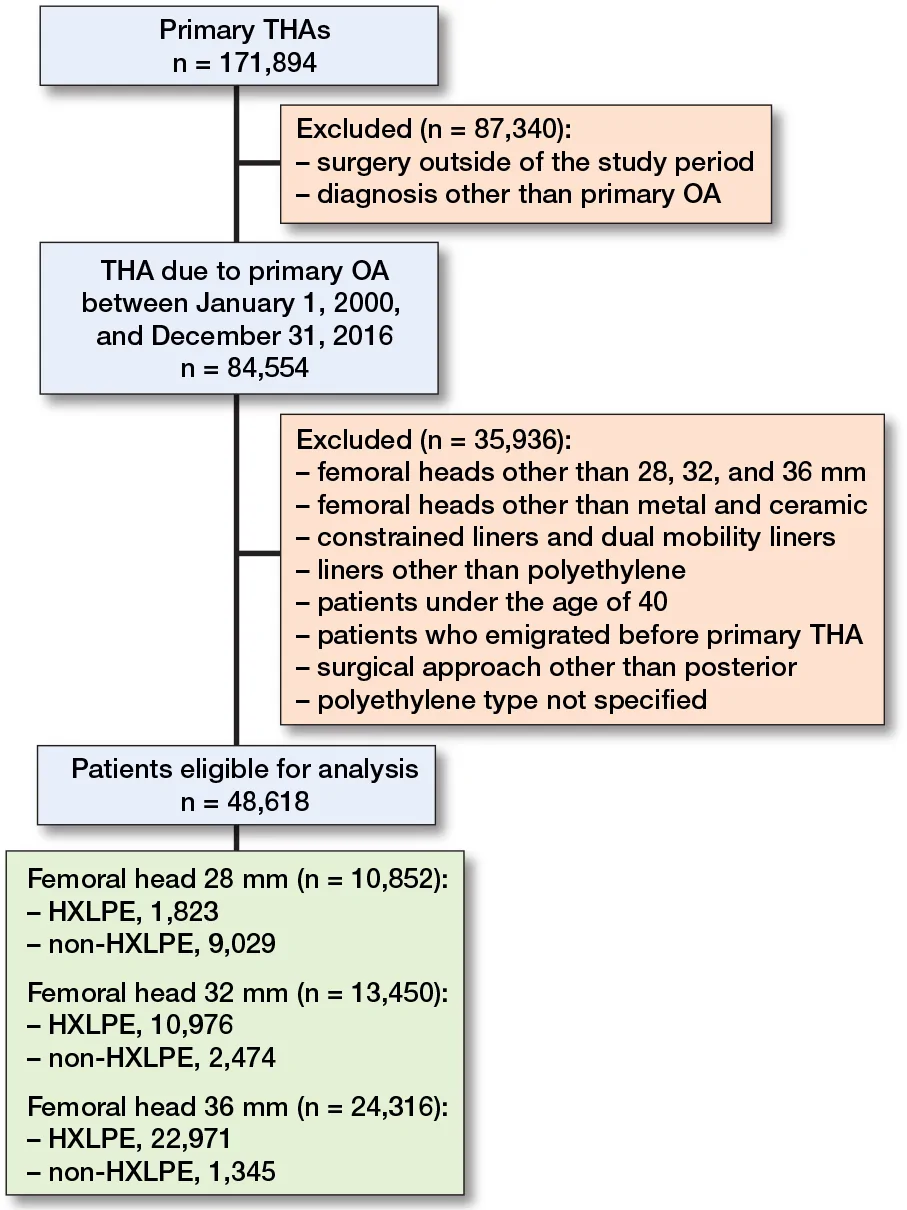
Flowchart illustrating participant flow.

48,618 patients were included in the study. The 36 mm group (n = 24,316) was the largest, followed by the 32 mm group (n = 13,450), and finally the 28 mm group (n = 10,852) (Figure 1). In the 28 mm group, most patients received non-HXLPE implants (83%), whereas most patients in the 32 mm and 36 mm groups received HXLPE implants (82% and 95%, respectively). Across all head-size groups, the majority of patients were older than 65 years. The majority of patients in all groups had a CCI score of 0. Among non-HXLPE groups, cemented fixation was predominant, whereas uncemented fixation was most common in the HXLPE groups (Tables 1 and 2). Unadjusted and standardized cumulative incidences are presented in Tables 3–6.

**Table 1:** Baseline characteristics of the population stratified by femoral head size. Values are count (%) or as specified

Item	Femoral head size		
	28 mm n = 10,852	32 mm n = 13,450	36 mm n = 24,316
Age at primary surgery (SD)	69 (9.6)	72 (8.9)	69 (8.9)
Age group			
40–65	3,438 (32)	3,720 (28)	7,506 (31)
65–75	4,053 (37)	5,328 (40)	10,461 (43)
> 75	3,361 (31)	4,402 (33)	6,349 (26)
Sex			
Female	6,607 (61)	9,064 (67)	12,101 (50)
Male	4,245 (39)	4,386 (33)	12,215 (50)
CCI			
0	7,288 (67)	8,633 (64)	16,029 (66)
1	2,849 (26)	3,660 (27)	6,484 (27)
2	715 (6.5)	1,157 (8.6)	1,803 (7.4)
Polyethylene type			
Non-HXLPE	9,029 (83)	2,474 (18)	1,345 (5.5)
HXLPE	1,823 (17)	10,976 (82)	22,971 (94)
Fixation			
Cemented	1,767 (16)	3,264 (24)	878 (3.6)
Uncemented	5,169 (48)	8,353 (62)	20,288 (83)
Hybrid	3,916 (36)	1,833 (14)	3,150 (13)
Head material			
Metal	8,048 (74)	11,452 (85)	22,621 (93)
Ceramic	2,804 (26)	1,998 (15)	1,695 (7.0)

CCI = Charlson comorbidity index; HXLPE = highly cross-linked polyethylene; Non-HXLPE = conventional polyethylene; SD = standard deviation.

**Table 2:** Baseline characteristics of the population stratified by femoral head size and polyethylene type. Values are count (%) or as specified

Item	28-mm femoral head		32-mm femoral head		36-mm femoral head	
	Non-HXLPE	HXLPE	Non-HXLPE	HXLPE	Non-HXLPE	HXLPE
	n = 9,029	n = 1,823	n = 2,474	n = 10,976	n = 1,345	n = 22,971
Potential follow-up, years (IQR)	16.7 (15.3–17.9)	15.0 (13.1–16.8)	12.7 (10.5–14.7)	10.3 (7.8–13.3)	8.1 (6.6–10.7)	8.3 (6.5–10.3)
Age at primary surgery (SD)	70 (9.5)	68 (9.8)	74 (8.4)	70 (9.4)	69 (8.4)	69 (9.4)
Age group						
40–65	2,681 (30)	757 (42)	377 (15)	3,343 (31)	384 (29)	7,122 (31)
65–75	3,399 (38)	654 (36)	944 (38)	4,384 (40)	663 (49)	9,798 (43)
> 75	2,949 (33)	412 (23)	1,153 (47)	3,249 (29)	298 (22)	6,051 (26)
Sex						
Female	5,403 (60)	1,204 (66)	1,635 (66)	7,429 (68)	620 (46)	11,481 (50)
Male	3,626 (40)	619 (34)	839 (34)	3,547 (32)	725 (54)	11,490 (50)
CCI						
0	6,062 (67)	1,226 (67)	1,503 (61)	7,130 (65)	860 (64)	15,169 (66)
1	2,369 (26)	480 (26)	695 (28)	2,965 (27)	363 (27)	6,121 (27)
2	598 (6.6)	117 (6.4)	276 (11)	881 (8.0)	122 (9.1)	1,681 (7.3)
Fixation						
Cemented	1,555 (17)	212 (12)	1,441 (58)	1,823 (17)	50 (3.7)	828 (3.6)
Uncemented	4,025 (45)	1,144 (63)	899 (36)	7,454 (68)	1,214 (90)	19,074 (83)
Hybrid	3,449 (38)	467 (26)	134 (5.4)	1,699 (16)	81 (6.0)	3,069 (13)
Head material						
Metal	6,862 (76)	1,186 (65)	2,232 (90)	9,220 (84)	1,189 (88)	21,432 (93)
Ceramic	2167 (24)	637 (35)	242 (10)	1,756 (16)	156 (12)	1,539 (6.7)

For abbreviations, see Table 1.

**Table 3:** Unstandardized cumulative incidence of revision due to all-causes, aseptic loosening, infection, and dislocation at 10 years with death as a competing risk stratified by femoral head size and polyethylene type

Endpoint Head size	Non-HXLPE			HXLPE		
	Number at risk	Number of events	Cumulative incidence, % (CI)	Number at risk	Number of events	Cumulative incidence, % (CI)
Revision due to all causes						
28 mm	6,170	19	7.9 (7.3–8.4)	1,214	≤ 5	8.7 (7.4–10.0)
32 mm	1,333	≤ 5	7.3 (6.3–8.3)	4,987	8	6.8 (6.3–7.3)
36 mm	376	≤ 5	7.9 (6.3–9.4	6,847	17	5.7 (5.4–6.0)
Revision due to aseptic loosening						
28 mm	6,167	≤ 5	0.6 (0.5–0.8)	1213	≤ 5	0.7 (0.3–1.0)
32 mm	1,333	≤ 5	1.3 (0.8–1.7)	4,987	≤ 5	0.6 (0.5–0.8)
36 mm	376	≤ 5	0.8 (0.3–1.3)	6,847	≤ 5	0.6 (0.5–0.7)
Revision due to infection						
28 mm	6,167	≤ 5	0.6 (0.4–0.7)	1,213	≤ 5	0.5 (0.2–0.9)
32 mm	1,333	≤ 5	0.6 (0.3–0.9)	4,987	≤ 5	0.6 (0.4–0.7)
36 mm	376	≤ 5	1.1 (0.6–1.7)	6,847	≤ 5	0.7 (0.6–0.8)
Revision due to dislocation						
28 mm	6,167	≤ 5	1.5 (1.3–1.8)	1,213	≤ 5	1.9 (1.2–2.5)
32 mm	1,333	≤ 5	1.0 (0.6–1.4)	4,987	≤ 5	1.2 (1.0–1.5)
36 mm	376	≤ 5	1.4 (0.8–2.0)	6,847	≤ 5	0.9 (0.8–1.0)

For abbreviations, see Table 1. CI = 95% confidence interval

**Table 4:** Unstandardized estimates of the cumulative incidence of dislocation at 10 years with death or implant removal as competing risks for the entire population stratified by femoral head size and polyethylene type

Endpoint Head size	Non-HXLPE			HXLPE		
	Number at risk	Number of events	Cumulative incidence, % (CI)	Number at risk	Number of events	Cumulative incidence, % (CI)
Dislocation						
28 mm	5,729	16	9.9 (9.3–10.6)	1,134	≤ 5	9.8 (8.4–11.2)
32 mm	1,273	≤ 5	6.7 (5.7–7.7)	4,761	7	7.5 (7.0–8.0)
36 mm	362	≤ 5	6.7 (5.2–8.2)	6,583	9	5.6 (5.3–6.0)

For abbreviations, see Table 1. CI = 95% confidence interval

### 10-year risk of all-cause revision

HXLPE in the 36 mm group showed a statistically significant reduction of –3.1% (CI –4.5 to –1.6) in all-cause revisions compared with non-HXLPE, while no significant difference was found for the 28 mm and 32 mm groups (see Table 5).

**Table 5:** Cumulative incidence of revision for all-causes, aseptic loosening, infection, and dislocation at 10 years with death or implant removal as competing risks standardized according to the age, sex, CCI, and fixation distribution in the population stratified by femoral head size

Endpoint Head size	Non-HXLPE	HXLPE	ATE, % (CI)
	Cumulative incidence, % (CI)	Cumulative incidence, % (CI)	
Revision due to all causes			
28 mm	8.0 (7.5–8.6)	8.1 (7.0–9.1)	0.4 (–1.2 to 1.3)
32 mm	7.7 (6.6–8.7)	6.6 (6.1–7.1)	–1.1 (–2.2 to 0.07)
36 mm	9.1 (7.6–10.5)	6.0 (5.6–6.3)	–3.1 (–4.5 to –1.6)
Revision due to aseptic loosening			
28 mm	0.6 (0.4–0.8)	0.6 (0.2–0.9)	–0.03 (–0.4 to 0.4)
32 mm	1.6 (1.3–1.9)	0.8 (0.6–0.9)	–0.8 (–1.1 to –0.5)
36 mm	0.5 (0.1–0.9)	0.6 (0.5–0.7)	0.08 (–0.3 to 0.5)
Revision due to infection			
28 mm	0.6 (0.4–0.7)	0.5 (0.3–0.8)	–0.06 (–0.4 to 0.2)
32 mm	0.5 (0.3–0.7)	0.6 (0.5–0.8)	0.1 (–0.2 to 0.4)
36 mm	1.7 (1.2–2.1)	0.8 (0.7–0.9)	–0.9 (–1.3 to –0.4)
Revision due to dislocation			
28 mm	1.6 (1.3–1.8)	1.8 (1.2–2.3)	0.2 (–0.4 to 0.8)
32 mm	1.7 (1.2–2.2)	1.4 (1.2–1.6)	–0.4 (–0.9 to 0.2)
36 mm	2.2 (1.7–2.8)	0.9 (0.8–1.1)	–1.3 (–1.9 to –0.7)

For abbreviations, see Table 1. CI = 95% confidence interval. ATE = average treatment effect.

### 10-year risk of revision due to aseptic loosening

HXLPE in the 32 mm group showed a statistically significant reduction of –0.8% (CI –1.1 to –0.5) in revision due to aseptic loosening compared with non-HXLPE. There were no significant differences for the 28 mm and 36 mm groups (see Table 5).

### 10-year risk of revision due to infection

HXLPE in the 36 mm group showed a statistically significant reduction of –0.9% (CI –1.3 to –0.4) in revision due to infection compared with non-HXPLE. No significant difference was found for the 28 mm and 32 mm groups (see Table 5).

### 10-year risk of revision due to dislocation

HXLPE in the 36 mm group showed a statistically significant reduction of –1.3% (CI –1.9 to –0.7) revision due to dislocation compared with non-HXLPE. No significant difference was found for the 28 mm and 32 mm groups (see Table 5).

### 10-year risk of dislocation

HXLPE showed a statistically significant reduction of dislocations, –1.9% (CI –3.1 to –0.6) for the 36 mm group and –1.4% (CI –2.6 to –0.2) for the 32 mm group, while no difference was found for the 28 mm group (see Table 6).

**Table 6:** Cumulative incidence of dislocation at 10 years with death or implant removal as competing risks standardized according to the age, sex, CCI, and fixation distribution in the population stratified by femoral head size

Endpoint Head size	Non-HXLPE	HXLPE	ATE, % (CI)
	Cumulative incidence, % (CI)	Cumulative incidence, % (CI)	
Dislocation			
28 mm	10 (9.4–10.6)	9.1 (8.0–10.4)	–0.8 (–2.2 to 0.5)
32 mm	9.2 (8.1–10.4)	7.8 (7.3–8.3)	–1.4 (–2.3 to –0.2)
36 mm	7.6 (6.4–8.8)	5.7 (5.4–6.0)	–1.9 (–3.1 to –0.6)

For abbreviations, see Table 1. CI = 95% confidence interval. ATE = average treatment effect.

## Discussion

We aimed to investigate the 10-year risk of all-cause revision, cause-specific revisions, and dislocation comparing HXLPE and non-HXLPE after primary THA, presenting results separately for 28 mm, 32 mm, and 36 mm femoral heads.

We found a significantly lower risk of all-cause revision, revision due to infection or dislocation, and dislocations treated with open or closed reduction for 36 mm femoral heads with an HXLPE liner. Additionally, 32 mm femoral heads with an HXLPE liner were associated with a lower risk of revision due to aseptic loosening and dislocation. As results are presented separately for each head size rather than in a single pooled model, direct comparisons across head sizes were not performed, and findings should be interpreted within each stratum independently.

These findings reinforce previous literature demonstrating a lower risk of revision with HXLPE compared with non-HXLPE [10,18,19]. Studies that did not find such differences were often limited by smaller sample sizes or narrow inclusion criteria, frequently focusing on single fixation types or head sizes [20-22]. In contrast, our study included a large cohort encompassing multiple fixation methods and head sizes.

Regarding dislocation, the 36 mm HXLPE group consistently showed the lowest dislocation risk across all analyses. After standardization, dislocation risk was lower across HXLPE groups in the 32 mm and 36 mm cohorts. Furthermore, there was a reduction in revision due to dislocation in the 36 mm HXLPE group. Although larger femoral heads are associated with increased polyethylene wear, the implementation of HXLPE has substantially mitigated this issue [23,24]. Therefore, the observed reduction in dislocations and revision due to dislocation is likely attributable not directly to HXLPE itself, but rather to its capacity to support larger femoral head use, which offers biomechanical advantages such as increased jump distance and greater range of motion, thus reducing dislocation rates [25,26]. Furthermore, given that dislocation represents the most frequent indication for revision surgery in Denmark, the ability of HXLPE to support the use of larger femoral heads likely contributes to the favorable outcomes for all-cause revision and revision due to dislocation with 36 mm HXLPE implants [3]. This may explain the absence of a difference between the 28 mm groups, as smaller femoral heads are less prone to PE wear and may therefore not benefit from HXLPE compared with non-HXLPE [27,28].

In the 36 mm HXLPE group, we observed a lower risk of revisions due to infection. While we were unable to identify clinical studies demonstrating a similar association, in vitro evidence indicates that HXLPE may inhibit bacterial and fungal adhesion [29]. The observed association between PE type and reduced infection-related revision most likely represents an incidental finding.

According to international hip arthroplasty registers, 32 mm femoral heads are currently the most commonly used head size [30]. Our findings suggest that, with the widespread adoption of HXLPE, 36 mm heads may offer better patient outcomes in the longer term, provided patient anatomy and surgical context permit their use. The combination of 36 mm heads with HXLPE demonstrated the most favorable outcomes for all-cause revision and dislocation, supporting a possible shift in clinical preference, particularly as non-HXLPE have become largely obsolete [3,19].

### Strengths

We used an algorithm validated in both the DHR and DNPR to identify all dislocations treated through both open and closed reduction, which is not routinely collected in registry data [31]. Furthermore, the NOMESCO codes used to identify outcomes are validated in the DNPR. In addition, the large sample sizes across all study groups enhanced the statistical power of our analyses. Finally, to minimize residual confounding and model misspecification bias, we standardized according to multiple potential confounders and employed a doubly robust method for inference on risk differences [15,16,32].

### Limitations

The DHR demonstrates a high national completeness rate of approximately 95% for revision surgeries [3]. However, the number of registrations for specific revision causes remains limited. Consequently, although the analyses of all-cause revision were supported by sufficient patient numbers and near-complete follow-up due to reliance on DNPR data, the cause-specific revision analyses were based on smaller samples, resulting in reduced statistical precision and wider 95% confidence intervals. Additionally, the absence of data on potential confounders such as body mass index, American Society of Anesthesiologists Physical Status Classification System score, activity level, and specific comorbidities that may influence liner wear or revision risk limited our ability to perform more comprehensive adjustments. These variables are either not routinely recorded in the DHR or contain substantial missing data, which may have contributed to residual confounding. Furthermore, given the observational study design, residual confounding cannot be excluded, and the associations observed should not be interpreted as direct causal effects of HXLPE. Finally, revisions were defined using NOMESCO procedure codes indicating removal, exchange, or secondary implantation of a bone-anchored component. Vancouver type C periprosthetic fractures may be treated with osteosynthesis alone, which was not captured in our definition; consequently, our study may underestimate the risk of all-cause revision.

### Conclusion

We observed that 36 mm femoral heads with HXLPE were associated with lower risk of dislocation, all-cause revision, and revision due to dislocation or infection, while 32 mm femoral heads with HXLPE were associated with lower risk of dislocation and revision due to aseptic loosening. While these findings are consistent with a potential benefit of HXLPE, the findings should be interpreted cautiously and do not establish a causal relationship given the observational nature of the study.

## Notes

MC: conceptualization, data curation, formal analysis, investigation, methodology, visualization, project administration, writing—original draft, writing—review and editing. AI: conceptualization, data curation, formal analysis, investigation, methodology, project administration, validation, writing—original draft, writing—review and editing. FE: methodology, validation, supervision, writing—original draft, writing—review and editing. MSS, SO: conceptualization, methodology, supervision, writing—review and editing. MMP: conceptualization, funding acquisition, methodology, supervision, writing—review and editing

Handling co-editors: Per Henrik Randsborg and Philippe Wagner

*Acta* thanks David F Hamilton and Peter Lawrence Lewis for help with peer review of this manuscript.

## Appendices

### Appendix 1: Case definitions of dislocation and revision

#### Dislocation

Dislocations were defined as the sum of unique occurrences of either true dislocations—International Classification of Diseases (ICD) 10 code T84.0(A) and Nordic Medico-Statistical Committee (NOMESCO) procedure code NHF20, with laterality matching that of the primary total hip arthroplasty (THA), or probable dislocations—1 of ICD 10 codes S73.0 or NOMESCO procedure codes NFH00, NFH02, NFH20, NFH21, NFH22, either with known laterality matching the primary THA or unknown laterality). For each patient, only the first event of either a true or probable dislocation was counted.

#### Revision

Revision surgery was identified in the Danish National Patient Register (DNPR) and defined as the first occurrence of NOMESCO procedure codes NFC2, NFC3, NFC4, NFU10, NFU11, NFU12, or NFU19, with laterality matching that of the primary THA.

### Appendix 2

1. PE type only.
2. All covariates.
3. PE type + age + sex + strata (fixation) + CCI.
4. PE type + age + sex + fixation + strata (CCI).
5. PE type + age + sex + strata (fixation) + strata (CCI).
